# 4-Terminal Inorganic Perovskite/Organic Tandem Solar Cells Offer 22% Efficiency

**DOI:** 10.1007/s40820-022-00995-2

**Published:** 2022-12-29

**Authors:** Ling Liu, Hanrui Xiao, Ke Jin, Zuo Xiao, Xiaoyan Du, Keyou Yan, Feng Hao, Qinye Bao, Chenyi Yi, Fangyang Liu, Wentao Wang, Chuantian Zuo, Liming Ding

**Affiliations:** 1 Center for Excellence in Nanoscience (CAS), Key Laboratory of Nanosystem and Hierarchical Fabrication (CAS), National Center for Nanoscience and Technology, Beijing, 100190 People’s Republic of China; 2 School of Metallurgy and Environment, Central South University, Changsha, 410083 People’s Republic of China; 3 School of Physics, State Key Laboratory of Crystal Materials, Shandong University, Jinan, 250100 People’s Republic of China; 4 School of Environment and Energy, South China University of Technology, Guangzhou, 510000 People’s Republic of China; 5 School of Materials and Energy, University of Electronic Science and Technology of China, Chengdu, 610054 People’s Republic of China; 6 School of Physics and Electronic Science, East China Normal University, Shanghai, 200241 People’s Republic of China; 7 State Key Laboratory of Power System, Department of Electrical Engineering, Tsinghua University, Beijing, 100084 People’s Republic of China; 8 School of Materials Science and Engineering, Zhejiang Sci-Tech University, Hangzhou, 310018 People’s Republic of China; 9 Key Laboratory of Semiconductor Materials Science, Beijing Key Laboratory of Low Dimensional Semiconductor Materials and Devices, Institute of Semiconductors, Chinese Academy of Sciences, Beijing, 100083 People’s Republic of China

**Keywords:** 4-Terminal tandem solar cells, Inorganic perovskite solar cells, Organic solar cells, Semitransparent, Drop-coating

## Abstract

After fast developing of single-junction perovskite solar cells and organic solar cells in the past 10 years, it is becoming harder and harder to improve their power conversion efficiencies. Tandem solar cells are receiving more and more attention because they have much higher theoretical efficiency than single-junction solar cells. Good device performance has been achieved for perovskite/silicon and perovskite/perovskite tandem solar cells, including 2-terminal and 4-terminal structures. However, very few studies have been done about 4-terminal inorganic perovskite/organic tandem solar cells. In this work, semi-transparent inorganic perovskite solar cells and organic solar cells are used to fabricate 4-terminal inorganic perovskite/organic tandem solar cells, achieving a power conversion efficiency of 21.25% for the tandem cells with spin-coated perovskite layer. By using drop-coating instead of spin-coating to make the inorganic perovskite films, 4-terminal tandem cells with an efficiency of 22.34% are made. The efficiency is higher than the reported 2-terminal and 4-terminal inorganic perovskite/organic tandem solar cells. In addition, equivalent 2-terminal tandem solar cells were fabricated by connecting the sub-cells in series. The stability of organic solar cells under continuous illumination is improved by using semi-transparent perovskite solar cells as filter.

**Supplementary Information:**

The online version contains supplementary material available at 10.1007/s40820-022-00995-2.

## Highlights

- 4-Terminal inorganic perovskite/organic tandem solar cells were made by using semi-transparent inorganic perovskite solar cells and narrow-bandgap organic solar cells as the sub-cells, yielding a power conversion efficiency of 22.34%, which is the highest efficiency for inorganic perovskite/organic tandem solar cells.
- Inorganic perovskite solar cells made by drop-coating (self-spreading) gave much higher power conversion efficiency than the cells made by spin-coating, enabling perovskite/organic tandem solar cells with higher efficiency.

**Supplementary Information:**

The online version contains supplementary material available at 10.1007/s40820-022-00995-2.

## Graphical abstract

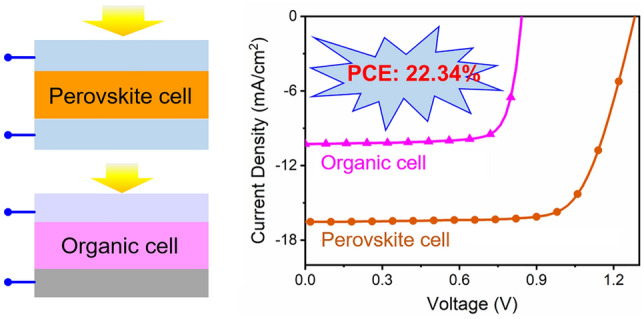

## Introduction

The power conversion efficiencies (PCE) of perovskite solar cells (PSC) and organic solar cells (OSC) increased very fast in the past decade. Certified PCEs of 25.7% [1] and 19.2% [2] have been achieved for single-junction PSC and OSC, respectively. As the PCEs getting closer to their theoretical limits, it is becoming harder and harder to further improve the PCE of single-junction PSC and OSC. Tandem solar cells are receiving increasing attentions because they have the potential to produce much higher PCEs than single-junction solar cells. Tandem solar cells can be divided into two types: two-terminal (2-T) and four-terminal (4-T) structures [3]. 2-T tandem cells are more popular due to their higher PCE. But it needs complicated equipment to make high-quality interconnecting layer, which is the key to make high-performance 2-T tandem cells [4]. For 4-T tandem cells, the two sub-cells are made separately by using common device fabrication equipment [5]. In addition, the device structure of the sub-cells in 4-T tandem cells can be different (e.g., one is* p*–*i*–*n* structure and the other is* n*–*i*–*p* structure). Another advantage of 4-T tandem cells is that the device performance is less susceptible to spectrum variation [4].

Both 2-T and 4-T perovskite-based tandem solar cells have been investigated, such as perovskite/silicon [6–8], perovskite/CIGS [9, 10], and perovskite/perovskite [11–13] tandem cells. The PCE of perovskite/organic solar cells legs behind the other perovskite-based tandem cells due to the lack of high-performance low-bandgap organic solar cells. Promoted by the fast-increasing efficiency of organic solar cells in the recent years, more and more people pay attention to perovskite/organic tandem solar cells. Compared with other perovskite-based tandem solar cells, perovskite/organic tandem solar cells possess low-temperature solution processing and light weight. A state-of-the-art PCE of 24.0% has been achieved for organic–inorganic hybrid perovskite/organic tandem solar cells [14]. Inorganic perovskite shows better thermal stability than organic–inorganic hybrid perovskite, but the PCE of inorganic perovskite/organic tandem solar cells is much lower than that of hybrid perovskite/organic tandem solar cells [15]. In 2019, we first reported 2-T inorganic perovskite/organic tandem solar cells [16], after which a series works were carried out [17–23]. A PCE of 21.4% has been achieved for 2-T inorganic perovskite/organic tandem cells [24]. To date, very few works have been done about 4-T inorganic perovskite/organic tandem cells. Li et al. fabricated 4-T tandem cells by using CsPbBr_3_ and PBDB-T-SF:IT-4F as the light-harvesting layers for the sub-cells, achieving a PCE of 14.03% [25]. There is still large room for the enhancement of PCE for 4-T inorganic perovskite/organic tandem cells.

In this work, we made 4-T inorganic perovskite/organic tandem solar cells by using semi-transparent inorganic PSC and D18-Cl-B:N3:PC_61_BM OSC as the sub-cells and investigated the relation between device performance and fabrication conditions. Equivalent 2-T tandem solar cells were also made by connecting the PSC and OSC in series. To obtain higher PCE, we use drop-coating instead of spin-coating to make more efficient inorganic perovskite films, achieving PCEs of 15.52% and 22.34% for semi-transparent inorganic PSC and 4-T perovskite/organic tandem solar cells, respectively.

## Experimental Section

### Solution Preparation

SnO_2_ colloidal dispersion (Alfa Aesar, 15 wt%) was diluted with deionized water in a volume ratio of 1:5 for the preparation of SnO_2_ layer. ZnO precursor solution was prepared by mixing 20 mg Zinc acetate dihydrate, 5.6 μL ethanolamine in 1 mL dimethoxy ethanol. PEDOT:PSS precursor solution was filtered with disposable hydrophilic filter (0.45 μm). The CsPbI_2_Br precursor solution was prepared by dissolving 0.8 M CsI, 0.4 M PbI_2_, and 0.4 M PbBr_2_ in* N,N*-dimethylformamide (DMF) and dimethyl sulfoxide (DMSO) mixed solvent (4:1,* v/v*). The CsPbI_2.25_Br_0.75_ precursor solution was prepared by dissolving 0.8 M CsI, 0.5 M PbI_2_, and 0.3 M PbBr_2_ in DMF:DMSO (4:1,* v/v*) mixed solvent. D18-Cl-B:N3:PC_61_BM solution with a weight ratio of 1:1.4:0.2 was dissolved in chloroform with a total concentration of 12.5 mg mL^−1^, diphenyl ether (DPE) was added into the solution as additive with a concentration of 0.5% (v/v). Solution for the hole transport layer of perovskite solar cells was prepared by dissolving PTAA and PBD2T (weight ratio 6:1) in chlorobenzene (CB) at a total concentration of 10 mg mL^−1^, and then stirred overnight at 40 °C. PDIN solution was prepared by mixing 2 mg PDIN and 3 μL acetic acid in 1 mL methanol.

### Materials Characterization

The thicknesses of the films were measured by using a KLA Tencor D-120 profilometer. Absorption spectra for the films were recorded on a Shimadzu UV-1800 spectrophotometer. Scanning electronic microscopy (SEM) images were taken with a Zeiss Merlin field emission SEM (FE-SEM) operated at an accelerating voltage of 5 kV. Atomic force microscopy (AFM) image was performed on Bruker Multimode-8 scanning probe microscope.

### Fabrication of Single-junction Perovskite Cells

Patterned ITO glass with a sheet resistance of 15 Ω sq^−1^ was cleaned by ultrasonic treatment in detergent, deionized water, acetone, isopropanol sequentially and then treated with UV-ozone for 10 min. SnO_2_ dispersion was spin-coated onto ITO glass at 3000 rpm for 30 s and then annealed at 150 °C in air for 30 min. ZnO precursor solution was spin-coated onto the SnO_2_ layer at 4000 rpm for 30 s and annealed at 200 °C in air for 20 min. Then the substrates were treated with UV-ozone for 5 min and transferred into a N_2_ glovebox. For perovskite films made by spin-coating, the perovskite precursor solution was spin-coated onto the substrates at 2000 rpm for 35 s, and annealed at 250 °C for 10 min. For perovskite films made by drop-coating, 1 μL solution was dropped onto the center of a 1.5 × 1.5 cm^2^ substrate which was preheated on a 60 °C hotplate, the solution can spread on the substrate spontaneously, producing a round film. The wet film was dried by N_2_ blowing and annealed at 250 °C for 10 min. HTL solution was then spin-coated onto the perovskite layer at 4000 rpm for 30 s, and annealed at 120 °C for 10 min. MoO_3_ (~ 6 nm) was evaporated onto the HTL through a shadow mask under vacuum (ca. 10^–4^ Pa). For the fabrication of opaque cells, 100 nm Ag was evaporated onto the MoO_3_ layer through a shadow mask under vacuum (ca. 10^–4^ Pa). For the fabrication of semi-transparent cells, 250 nm ITO was sputtered onto the MoO_3_ layer by using a magnetron sputtering system.

### Fabrication of Single-junction Organic Cells

A 30 nm thick PEDOT:PSS layer was made by spin-coating an aqueous dispersion onto ITO glass at 4000 rpm for 30 s. PEDOT:PSS substrates were dried at 150 °C for 10 min. The D18-Cl-B:N3:PC_61_BM solution was spin-coated onto PEDOT:PSS layer. PDIN solution was spin-coated onto the D18-Cl-B:N3:PC_61_BM layer at 5000 rpm for 30 s. Ag (~ 80 nm) was evaporated onto PDIN through a shadow mask (pressure ca. 10^–4^ Pa).

### Device Measurements

The illumination intensity was determined by using a monocrystalline silicon solar cell (Enli SRC 2020, 2 × 2 cm^2^) calibrated by NIM. The effective area for the devices is 4 mm^2^. *J*–*V* curves were measured by using a computerized Keithley 2400 SourceMeter and a Xenon-lamp-based solar simulator (Enli Tech, AM 1.5G, 100 mW cm^−2^). For the measurement of the filtered OSC, semi-transparent PSC with an area of ~ 1 cm^2^ was used as the filter. The PCE for the filtered OSC was measured by putting the OSC behind the filter. The external quantum efficiency (EQE) spectra were measured by using a QE-R3011 measurement system (Enli Tech).

## Results and Discussion

**Fig. 1:**
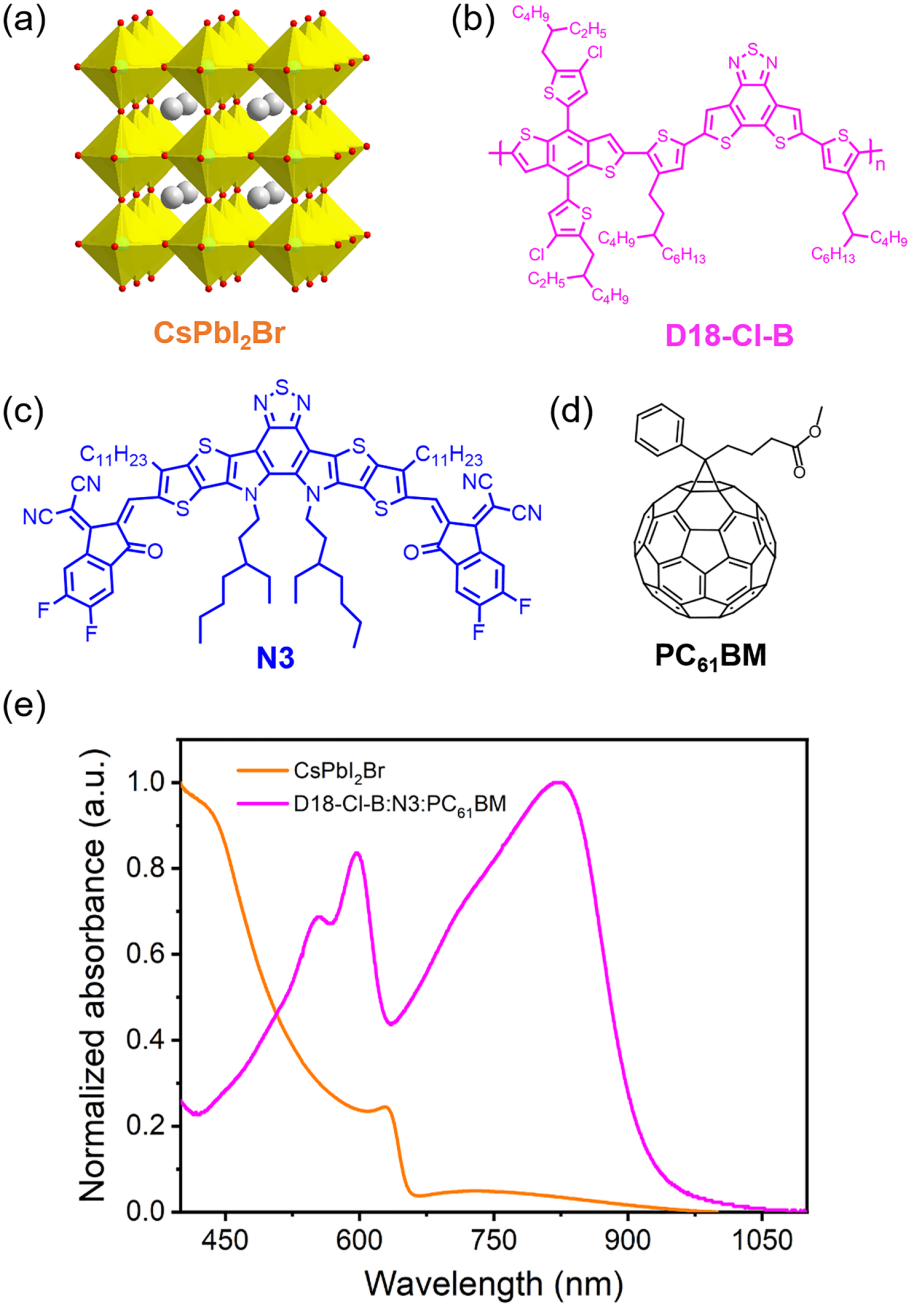
**a** Crystal structure of CsPbI_2_Br. **b–d** chemical structures for D18-Cl-B, N3, and PC_61_BM. **e** UV–Vis absorption spectra for CsPbI_2_Br and D18-Cl-B:N3:PC_61_BM (1:1.4:0.2) films

We first employed CsPbI_2_Br (Fig. 1a) as the light-harvesting layer for wide-bandgap inorganic PSC due to its suitable bandgap and good stability [26, 27]. The active layer for the narrow-bandgap OSC is composed of a wide-bandgap polymer D18-Cl-B (Eg^opt^ = 1.98 eV) [28] (Fig. 1b), a narrow-bandgap non-fullerene molecule N3 (Eg^opt^ = 1.32 eV) [29] (Fig. 1c), and PC_61_BM (Fig. 1d). The CsPbI_2_Br film shows a light absorption onset at 650 nm and a shoulder peak at 628 nm (Fig. 1e). The D18-Cl-B:N3:PC_61_BM film shows relatively low absorbance for visible light, with a strong absorption peak at 822 nm and an absorption onset at 946 nm. The light absorption spectra of CsPbI_2_Br and D18-Cl-B:N3:PC_61_BM films show good complementarity.

**Fig. 2:**
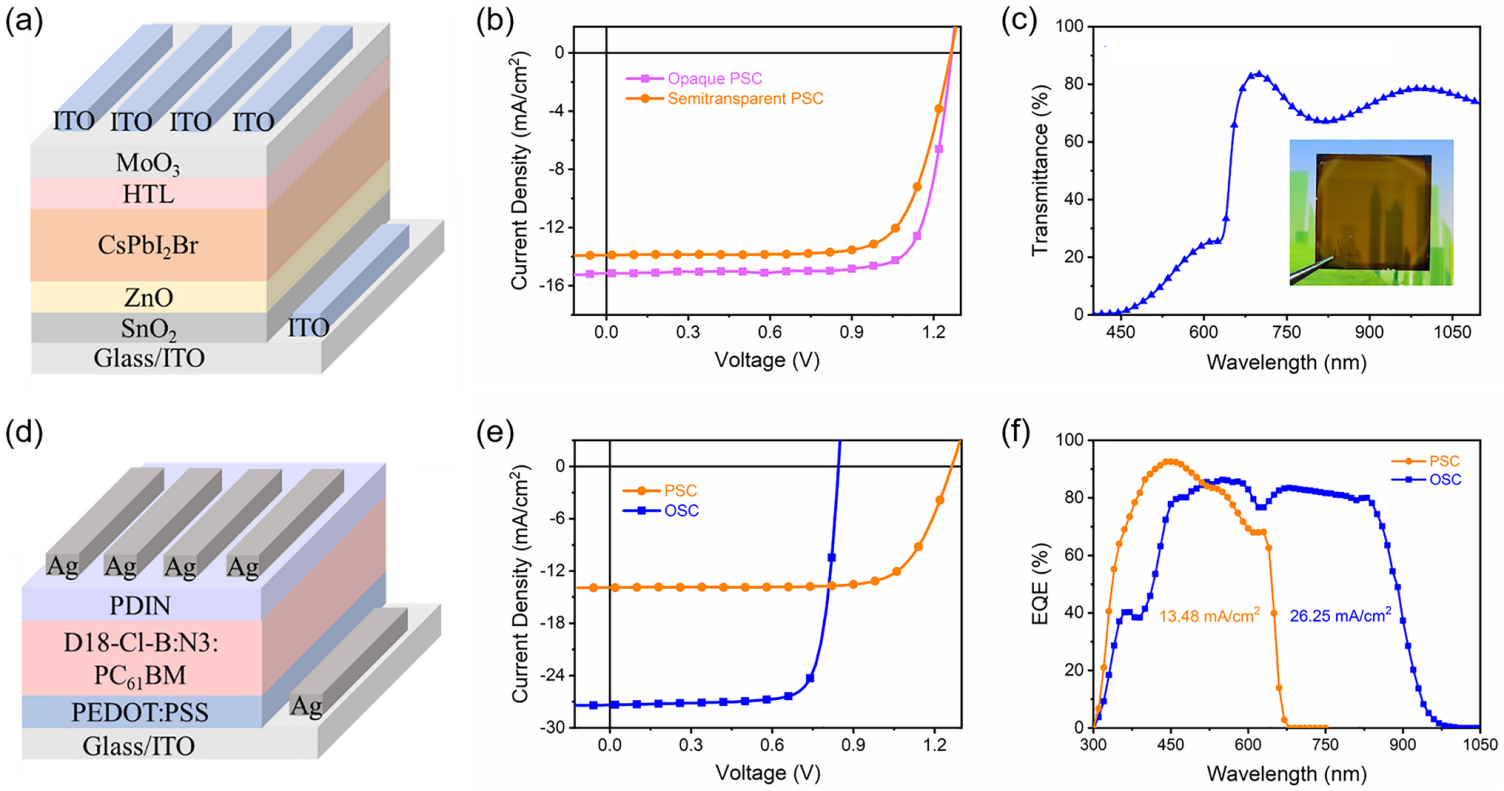
**a** structure of the semi-transparent CsPbI_2_Br solar cells. **b** *J–V* curves for the opaque and semi-transparent CsPbI_2_Br solar cells. **c** Transmittance spectrum for the semi-transparent CsPbI_2_Br solar cell. The inset shows a photo for the semi-transparent CsPbI_2_Br solar cell. **d** structure of the organic solar cells. **e** *J–V* curves for the semi-transparent CsPbI_2_Br solar cells and organic solar cells. **f** EQE spectra for the semi-transparent CsPbI_2_Br solar cells and organic solar cells

For tandem solar cells, the front cell should have good transmittance for long-wavelength light to ensure sufficient light reaches the rear cell. We made semi-transparent PSC by using sputtered ITO as the electrode instead of opaque Ag electrode (Fig. 2a). ITO with a thickness of 250 nm was used to obtain good transmittance and sufficient conductivity. The transmittance of the sputtered ITO above 680 nm exceeds 90% (Fig. S1). The thickness of the perovskite layer was optimized to maximize PCE of the semi-transparent PSC (Table S1), resulting in a best PCE of 12.99%, with an open-circuit voltage (*V*_oc_) of 1.26 V, a short-circuit current density (*J*_sc_) of 13.90 mA cm^−2^, and a fill factor (FF) of 73.99% (Table 1 and Fig. 2b). The average transmittance from 680 to 1100 nm for the whole semi-transparent PSC is 74.6% (Fig. 2c). The semi-transparent cell shows slightly lower PCE than the corresponding opaque cell due to lower *J*_sc_. The reduction in *J*_sc_ may be caused by the lower reflectance of ITO than that of Ag electrode, leading to reduced external quantum efficiency (EQE) near the band edge, where the light absorption is relatively weak (Fig. S2). The best single-junction OSC shows a PCE of 18.17%, with a *V*_oc_ of 0.84 V, a *J*_sc_ of 27.37 mA cm^−2^, and a FF of 78.60% (Fig. 2e, Table 1 and Table S2). The semi-transparent PSC exhibits a photoresponse range up to 680 nm, with an integrated current of 13.48 mA cm^−2^ (Fig. 2f). The OSC shows much wider photoresponse range (300–975 nm), yielding an integrated current of 26.25 mA cm^−2^. The PSC shows much higher external quantum efficiency (EQE) than the OSC below 500 nm, which means more high-energy photons can be harvested by using PSC.

**Table 1:** Performance data for opaque and semi-transparent CsPbI_2_Br perovskite solar cells, stand-alone and filtered organic solar cells, and 4-T tandem solar cell

Device	*V*_oc_ (V)	*J*_sc_ (mA cm^−2^)	FF (%)	PCE (%)
Opaque PSC	1.27	15.13	79.13	15.16
Semi-transparent PSC	1.26	13.90	73.99	12.99
Stand-alone OSC	0.84	27.37	78.60	18.17
Filtered OSC	0.82	12.66	79.35	8.26
4-terminal tandem cell	–	–	–	21.25

**Fig. 3:**
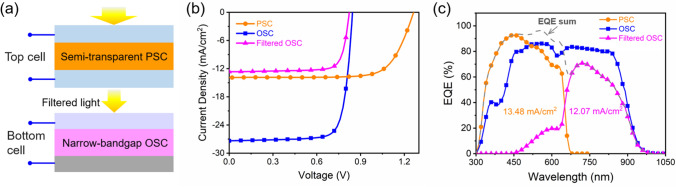
**a** Structure for the 4-terminal perovskite/organic tandem solar cells. **b** *J–V* curves for the best semi-transparent PSC (top cell), stand-alone OSC, and filtered OSC (bottom cell). **c** EQE spectra for the semi-transparent PSC, stand-alone OSC, and filtered OSC. The dash line is the sum of the EQE for the PSC and filtered OSC

To make 4-T tandem cells, the semi-transparent PSC was put onto the OSC (Fig. 3a). The PCE of the tandem cell is equal to the sum of the PCE for the two sub-cells, which were measured independently. CsPbI_2_Br cells made by using different conditions were used as filter to maximize the PCE of the filtered OSC, which yields a best PCE of 8.26% (Fig. 3b, Tables S3 and S4). The semi-transparent PSC and filtered OSC produce a total PCE of 21.25% for the 4-terminal tandem cells (Table 1). The semi-transparent PSC and filtered OSC show integrated currents of 13.48 and 12.07 mA cm^−2^, respectively (Fig. 3c). Compared with single-junction OSC, the tandem cell shows higher EQE below 645 nm but lower EQE above 645 nm, resulting in similar integrated currents. The higher PCE for the tandem cell is mainly contributed by the high photovoltage from the PSC.

**Fig. 4:**
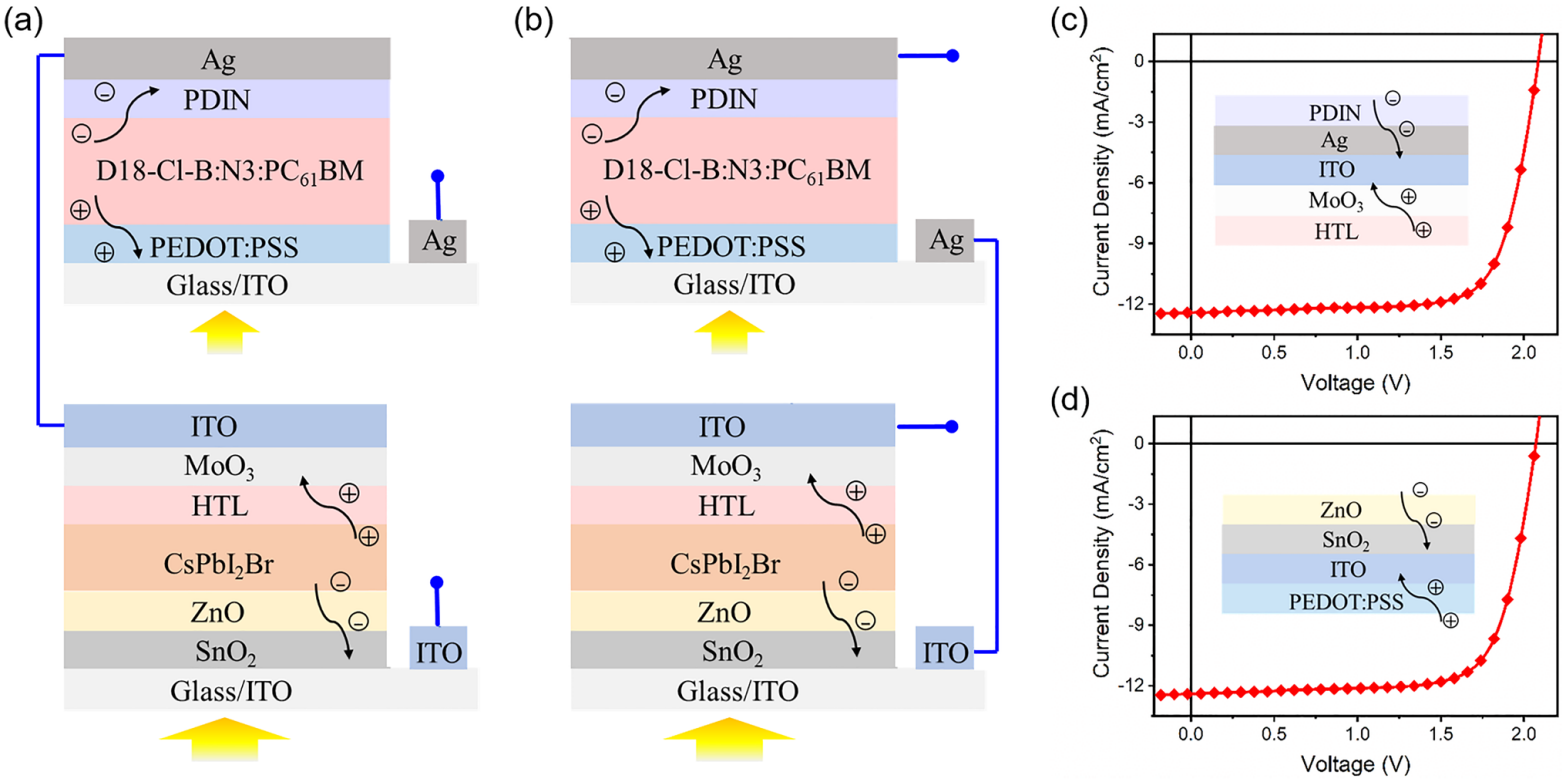
**a, b** Illustration of the two connecting methods to make the equivalent 2-terminal tandem solar cells. **c** and **d** *J–V* curves for the tandem cells shown in **a** and **b**. The insets show the corresponding interconnecting layers

As a comparison, equivalent 2-T tandem cells were made by connecting the champion semi-transparent PSC and OSC in series (Fig. 4). There are two connecting methods to make 2-T tandem cells, as shown in Fig. 4a, b. The 2-T tandem cells shown in Fig. 4a, b produce PCEs of 19.18% and 18.83%, respectively (Fig. 4 and Table 2). Considering that the light distribution in the sub-cells is the same for the two types of tandem cells, the difference in PCE may be caused by the difference in interconnecting layers (ICL), which are HTL/MoO_3_/ITO/Ag/PDIN and PEDOT:PSS/ITO/SnO_2_/ZnO for the tandem cells shown in Fig. 4a, b, respectively. The higher PCE for the cell in Fig. 4a implies that HTL/MoO_3_/ITO/Ag/PDIN is a better choice to be used as ICL in tandem cells. The *J*_sc_ of the 2-T tandem cells is slightly lower than the *J*_sc_ of the sub-cells (Table 1). The lower PCE of the 2-T structure compared with the 4-T structure is caused by mismatch of the photocurrents for the sub-cells and energy loss in the ICL.

**Table 2:** Performance data for 2-T tandem solar cells made by using different connecting methods

Connecting method	*V*_oc_ (V)	*J*_sc_ (mA cm^−2^)	FF (%)	PCE (%)
Figure 4a	2.08	12.42	74.06	19.18
Figure 4b	2.07	12.40	73.31	18.83

**Table 3:** Performance data for opaque and semi-transparent CsPbI_2.25_Br_0.75_ solar cells made by drop-coating, filtered OSC, and 4-T tandem cell

Device	*V*_oc_ (V)	*J*_sc_ (mA cm^−2^)	FF (%)	PCE (%)
Opaque PSC	1.29	16.87	80.46	17.47
Semi-transparent PSC	1.28	16.52	73.25	15.52
Filtered OSC	0.84	10.27	78.87	6.82
4-T tandem cell				22.34

**Fig. 5:**
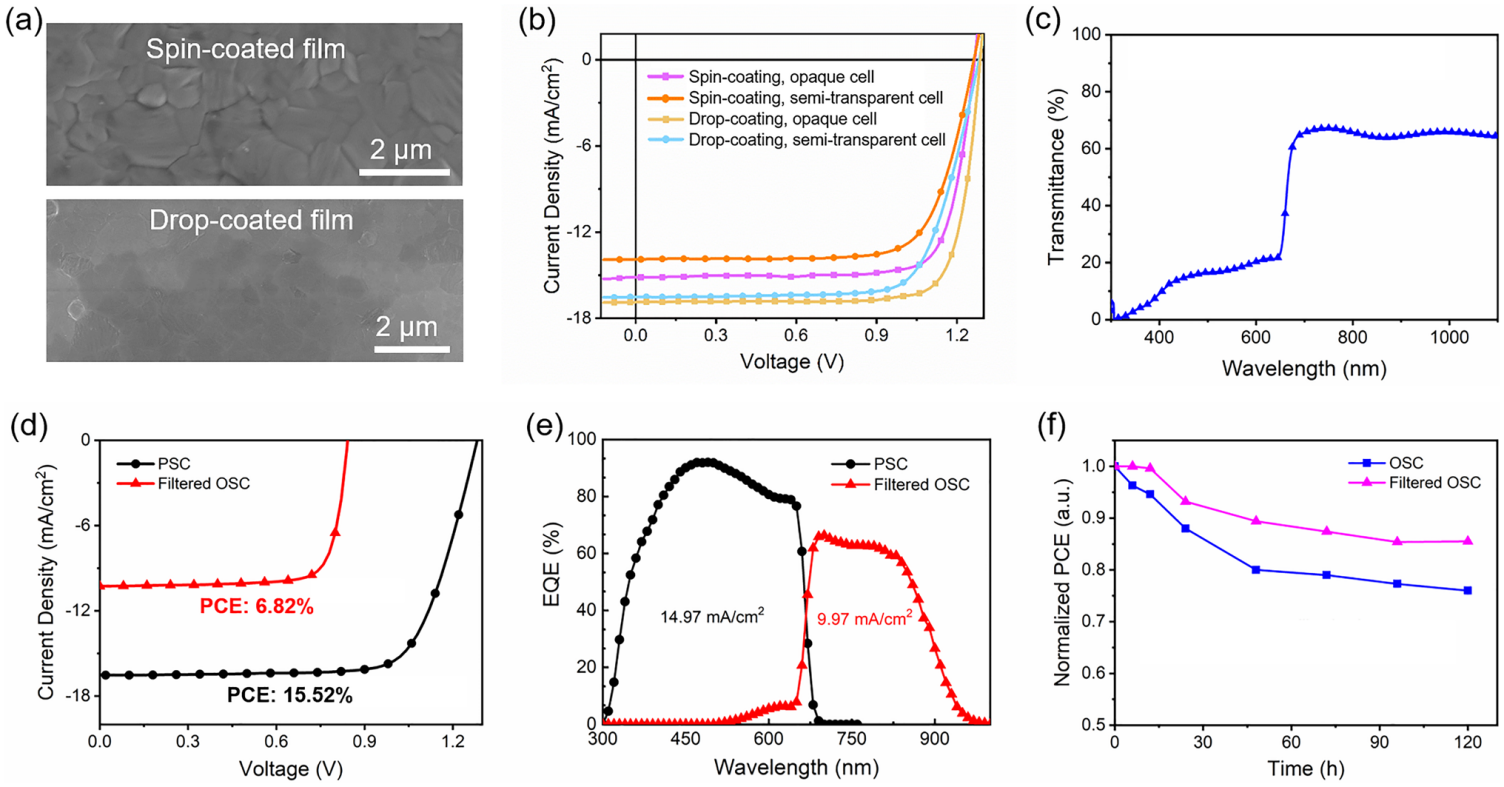
**a** SEM images for inorganic perovskite films made by spin-coating and drop-coating. **b** *J–V* curves for opaque and semi-transparent CsPbI_2.25_Br_0.75_ solar cells made by spin-coating and drop-coating. **c** Transmittance spectrum for the semi-transparent CsPbI_2.25_Br_0.75_ solar cell. **d** and **e** *J–V* curves and EQE spectra for the semi-transparent CsPbI_2.25_Br_0.75_ cell and filtered organic (D18-Cl-B:N3:PC_61_BM) cell. **f** PCE change for stand-alone OSCs and filtered OSCs under continuous 1-sun illumination

The above used CsPbI_2_Br films were made by spin-coating. Recently, we developed a modified drop-coating method (also known as self-spreading method) to make perovskite solar cells [30–36]. CsPbI_2_Br films made by drop-coating show better photovoltaic performance than the films made by spin-coating [34]. To further improve the PCE of inorganic PSC, we employed CsPbI_2.25_Br_0.75_ film made by drop-coating as the perovskite layer. The CsPbI_2.25_Br_0.75_ film shows more compact and uniform surface (Figs. 5a and S3), lower roughness (Fig. S4), and slightly lower bandgap than CsPbI_2_Br film (Fig. S5). PCEs of 17.47% and 15.52% were achieved for opaque and semi-transparent cells, respectively, which are much higher than the CsPbI_2_Br cells made by spin-coating (Fig. 5b). The enhanced PCE for the cells made by drop-coating is mainly due to the improved morphology of the perovskite films. The semi-transparent CsPbI_2.25_Br_0.75_ cell also shows lower EQE than that of the opaque cell (Fig. S6). The semi-transparent CsPbI_2.25_Br_0.75_ cell show an average transmittance of 65.4% above 690 nm (Fig. 5c). The OSC under light filtered by the CsPbI_2.25_Br_0.75_ cell show a PCE of 6.82% (Fig. 5d), resulting in a total PCE of 22.34% for 4-T tandem solar cell. Compared with the CsPbI_2_Br cell, the CsPbI_2.25_Br_0.75_ cell show higher integrated current (Fig. 5e), which is due to the broadened photoresponse (Table 3).

The stability of OSC under continuous one sun illumination was investigated. The PCEs maintain 76 and 86% of the initial values after 120 h illumination for the cells without and with PSC filter, respectively (Fig. 5f). The reduction in PCE is mainly attributed to the decrease in the fill factor (Fig. S7). The main reason is that organic solar cells are sensitive to ultraviolet light, which can break chemical bonds and cause photochemical reactions in the active layer of organic solar cells [20]. In tandem cells, the perovskite front cell act as a UV filter, thus reducing the influence of UV light on performance of organic solar cells.

## Conclusions

In summary, 4-T inorganic perovskite/organic tandem solar cells were made by using semi-transparent inorganic perovskite top cell and D18-Cl-B:N3:PC_61_BM organic bottom cell. Semi-transparent inorganic perovskite solar cells, organic solar cells under filtered light, and 4-T tandem cells show PCEs of 12.99, 8.26, and 21.25%, respectively. Equivalent 2-T tandem solar cells made by connecting the sub-cells in series show lower PCE due to the mismatch of photocurrent of the sub-cells. Besides performance of sub-cells, connecting methods of the sub-cells also affect the device performance of 2-T tandem solar cells, which may be due to the difference in interconnecting layers. By using drop-coating instead of spin-coating to make the inorganic perovskite films, the PCE of semi-transparent inorganic PSC was enhanced to 15.52%, boosting the PCE of 4-T tandem solar cells to 22.34%, which is much higher than the PCE of the reported 4-T perovskite/organic tandem solar cells, and also higher than that of the 2-T inorganic perovskite/organic tandem solar cells. Moreover, the stability of the OSC under continuous illumination was improved because the UV light is filtered by the perovskite cells. The performance of 4-T perovskite/organic tandem solar cells can be further improved by improving the PCE of the sub-cells and the transmittance of the perovskite sub-cell.

### Supplementary Information

Below is the link to the electronic supplementary material.

Supplementary file1 (PDF 566 KB)

## References

[1] Dong W, Qiao W, Xiong S, Yang J, Wang X (2022). Surface passivation and energetic modification suppress nonradiative recombination in perovskite solar cells. Nano-Micro Lett..

[2] Zhu L, Zhang M, Xu J, Li C, Yan J (2022). Single-junction organic solar cells with over 19% efficiency enabled by a refined double-fibril network morphology. Nat. Mater..

[3] Fang Z, Zeng Q, Zuo C, Zhang L, Xiao H (2021). Perovskite-based tandem solar cells. Sci. Bull..

[4] Fu F, Feurer T, Jäger T, Avancini E, Bissig B (2015). Low-temperature-processed efficient semi-transparent planar perovskite solar cells for bifacial and tandem applications. Nat. Commun..

[5] Zhu Z, Mao K, Xu J (2021). Perovskite tandem solar cells with improved efficiency and stability. J. Energy Chem..

[6] Al-Ashouri A, Köhnen E, Li B, Magomedov A, Hempel H (2020). Monolithic perovskite/silicon tandem solar cell with >29% efficiency by enhanced hole extraction. Science.

[7] Wang Z, Zhu X, Zuo S, Chen M, Zhang C (2020). 27%-efficiency four-terminal perovskite/silicon tandem solar cells by sandwiched gold nanomesh. Adv. Funct. Mater..

[8] Chen Y, Ying Z, Li X, Wang X, Wu J (2022). Self-sacrifice alkali acetate seed layer for efficient four-terminal perovskite/silicon tandem solar cells. Nano Energy.

[9] Zhao P, Feng L, Lin Z, Wang J, Su J (2019). Theoretical analysis of two-terminal and four-terminal perovskite/copper indium gallium selenide tandem solar cells. Solar RRL.

[10] Zuo S, Chu S, An P, Hu H, Yin Z (2021). Solvent coordination engineering for high-quality hybrid organic-inorganic perovskite films. J. Mater. Sci..

[11] K. Xiao, R. Lin, Q. Han, Y. Hou, Z. Qin et al., All-perovskite tandem solar cells with 24.2% certified efficiency and area over 1 cm^2^ using surface-anchoring zwitterionic antioxidant. Nat. Energy **5**(11), 870–880 (2020). 10.1038/s41560-020-00705-5

[12] Yoon S, Ha HU, Seok H-J, Kim H-K, Kang D-W (2022). Highly efficient and reliable semitransparent perovskite solar cells via top electrode engineering. Adv. Funct. Mater..

[13] Zhao D, Wang C, Song Z, Yu Y, Chen C (2018). Four-terminal all-perovskite tandem solar cells achieving power conversion efficiencies exceeding 23%. ACS Energy Lett..

[14] Brinkmann KO, Becker T, Zimmermann F, Kreusel C, Gahlmann T (2022). Perovskite-organic tandem solar cells with indium oxide interconnect. Nature.

[15] Xie Y-M, Xue Q, Yao Q, Xie S, Niu T (2021). Monolithic perovskite/organic tandem solar cells: Developments, prospects, and challenges. Nano Select..

[16] Zeng Q, Liu L, Xiao Z, Liu F, Hua Y (2019). A two-terminal all-inorganic perovskite/organic tandem solar cell. Sci. Bull..

[17] Lang K, Guo Q, He Z, Bai Y, Yao J (2020). High performance tandem solar cells with inorganic perovskite and organic conjugated molecules to realize complementary absorption. J. Phys. Chem. Lett..

[18] Aqoma H, Imran IF, Wibowo FTA, Krishna NV, Lee W (2020). High-efficiency solution-processed two-terminal hybrid tandem solar cells using spectrally matched inorganic and organic photoactive materials. Adv. Energy Mater..

[19] Xie S, Xia R, Chen Z, Tian J, Yan L (2020). Efficient monolithic perovskite/organic tandem solar cells and their efficiency potential. Nano Energy.

[20] Chen X, Jia Z, Chen Z, Jiang T, Bai L (2020). Efficient and reproducible monolithic perovskite/organic tandem solar cells with low-loss interconnecting layers. Joule.

[21] Wang P, Li W, Sandberg OJ, Guo C, Sun R (2021). Tuning of the interconnecting layer for monolithic perovskite/organic tandem solar cells with record efficiency exceeding 21%. Nano Lett..

[22] X. Gu, X. Lai, Y. Zhang, T. Wang, W. L. Tan et al., Organic solar cell with efficiency over 20% and V_oc_ exceeding 2.1 V enabled by tandem with all-inorganic perovskite and thermal annealing-free process. Adv. Sci. **9**(28), 2270178 (2022). 10.1002/advs.202270178 10.1002/advs.202200445 PMC9534952 35876031

[23] Chen W, Li D, Chen X, Chen H, Liu S (2022). Surface reconstruction for stable monolithic all-inorganic perovskite/organic tandem solar cells with over 21% efficiency. Adv. Funct. Mater..

[24] Y. Ding, Q. Guo, Y. Geng, Z. Dai, Z. Wang et al., A low-cost hole transport layer enables CsPbI_2_Br single-junction and tandem perovskite solar cells with record efficiencies of 17.8% and 21.4%. Nano Today **46**, 101586 (2022). 10.1016/j.nantod.2022.101586

[25] W. Chen, J. Zhang, G. Xu, R. Xue, Y. Li et al., A semitransparent inorganic perovskite film for overcoming ultraviolet light instability of organic solar cells and achieving 14.03% efficiency. Adv. Mater. **30**(21), e1800855 (2018). 10.1002/adma.201800855 10.1002/adma.201800855 29633397

[26] Xu J, Cui J, Yang S, Han Y, Guo X (2021). Unraveling passivation mechanism of imidazolium-based ionic liquids on inorganic perovskite to achieve near-record-efficiency CsPbI_2_Br solar cells. Nano-Micro Lett..

[27] S. Fu, X. Li, L. Wan, W. Zhang, W. Song et al., Effective surface treatment for high-performance inverted CsPbI_2_Br perovskite solar cells with efficiency of 15.92%. Nano-Micro Lett. **12**, 170 (2020). 10.1007/s40820-020-00509-y 10.1007/s40820-020-00509-y PMC7770732 34138163

[28] X. Meng, K. Jin, Z. Xiao, L. Ding, Side chain engineering on D18 polymers yields 18.74% power conversion efficiency. J. Semicond. **42**(10), 100501 (2021). 10.1088/1674-4926/42/10/100501

[29] Jiang K, Wei Q, Lai JYL, Peng Z, Kim HK (2019). Alkyl chain tuning of small molecule acceptors for efficient organic solar cells. Joule.

[30] Zuo C, Scully AD, Vak D, Tan W, Jiao X (2019). Self-assembled 2D perovskite layers for efficient printable solar cells. Adv. Energy Mater..

[31] Zuo C, Scully AD, Tan WL, Zheng F, Ghiggino KP (2020). Crystallisation control of drop-cast quasi-2D/3D perovskite layers for efficient solar cells. Commun. Mater..

[32] Zuo C, Scully AD, Gao M (2021). Drop-casting method to screen ruddlesden-popper perovskite formulations for use in solar cells. ACS Appl. Mater. Interfaces.

[33] Zuo C, Ding L (2021). Drop-casting to make efficient perovskite solar cells under high humidity. Angew. Chem. Int. Ed..

[34] Xiao H, Zuo C, Liu F, Ding L (2021). Drop-coating produces efficient CsPbI_2_Br solar cells. J. Semicond..

[35] Liu L, Zuo C, Ding L (2021). Self-spreading produces highly efficient perovskite solar cells. Nano Energy.

[36] Zhang L, Zuo C, Ding L (2021). Efficient MAPbI_3_ solar cells made via drop-coating at room temperature. J. Semicond..

