# Supplementary material for: 4-Terminal Inorganic Perovskite/Organic Tandem Solar Cells Offer 22% Efficiency
Source: Nanomicro Lett. 2022 Dec 29;15:23. doi: 10.1007/s40820-022-00995-2 (PMC9800665; doi:10.1007/s40820-022-00995-2)
Supplement: Supplementary file 1 — Supplementary file1 (PDF 566 KB) [file 40820_2022_995_MOESM1_ESM.pdf]

Nano-Micro Letters

Supporting Information for

4-Terminal Inorganic Perovskite/Organic Tandem Solar Cells Offer 22% Efficiency

Ling Liu[1, #], Hanrui Xiao[1, 2, #], Ke Jin[1], Zuo Xiao[1], Xiaoyan Du[3], Keyou Yan[4], Feng Hao[5], Qinye Bao[6], Chenyi Yi[7], Fangyang Liu[2, *], Wentao Wang[8, *], Chuantian Zuo[1, 9, *], and Liming Ding[1, *]

[1]Center for Excellence in Nanoscience (CAS), Key Laboratory of Nanosystem and Hierarchical Fabrication (CAS), National Center for Nanoscience and Technology, Beijing 100190, P. R. China

[2]School of Metallurgy and Environment, Central South University, Changsha 410083, P. R. China

[3]School of Physics, State Key Laboratory of Crystal Materials, Shandong University, Jinan 250100, P. R. China

[4]School of Environment and Energy, South China University of Technology, Guangzhou 510000, P. R. China

[5]School of Materials and Energy, University of Electronic Science and Technology of China, Chengdu 610054, P. R. China

[6]School of Physics and Electronic Science, East China Normal University, Shanghai 200241, P. R. China

[7]State Key Laboratory of Power System, Department of Electrical Engineering, Tsinghua University, Beijing 100084, P. R. China

[8]School of Materials Science and Engineering, Zhejiang Sci-Tech University, Hangzhou 310018, P. R. China

[9]Key Laboratory of Semiconductor Materials Science, Beijing Key Laboratory of Low Dimensional Semiconductor Materials and Devices, Institute of Semiconductors, Chinese Academy of Sciences, Beijing, 100083, P. R. China

[#]Ling Liu and Hanrui Xiao contributed equally to this work.

* Corresponding authors. E-mail: ding@nanoctr.cn (Liming Ding) ; zuocht@nanoctr.cn (Chuantian Zuo); liufangyang@csu.edu.cn (Fangyang Liu); wtwang@zstu.edu.cn (Wentao Wang)

Supplementary Figures and Tables

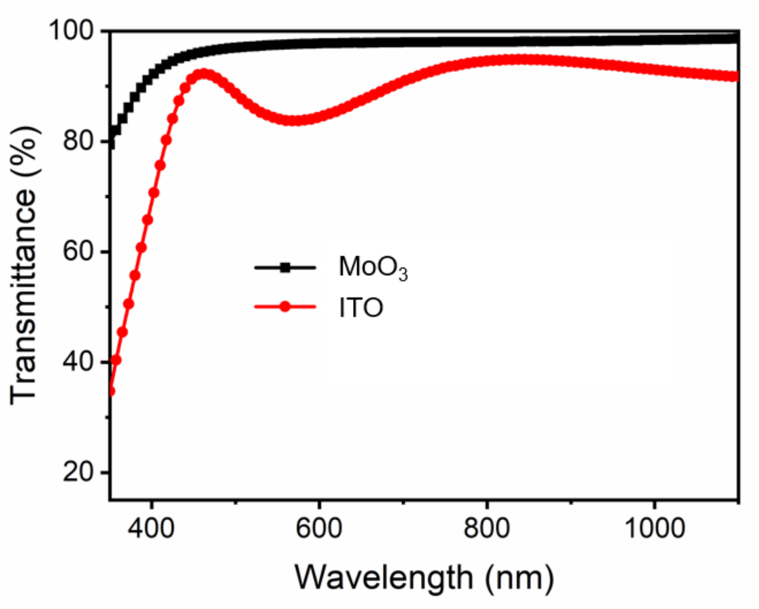

Fig. S1 Transmittance spectra for MoO_3 and sputtered ITO

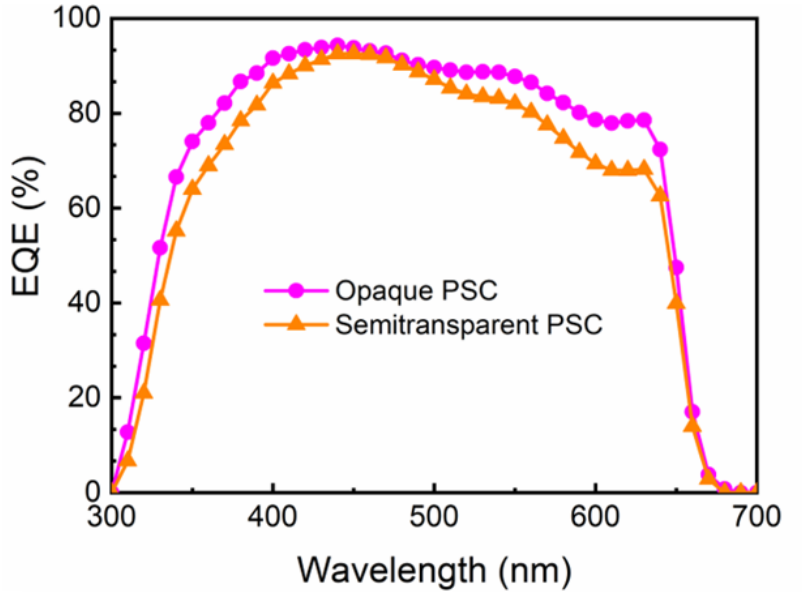

Fig. S2 EQE spectra for the opaque and semi-transparent $CsPbI_2Br$ solar cells

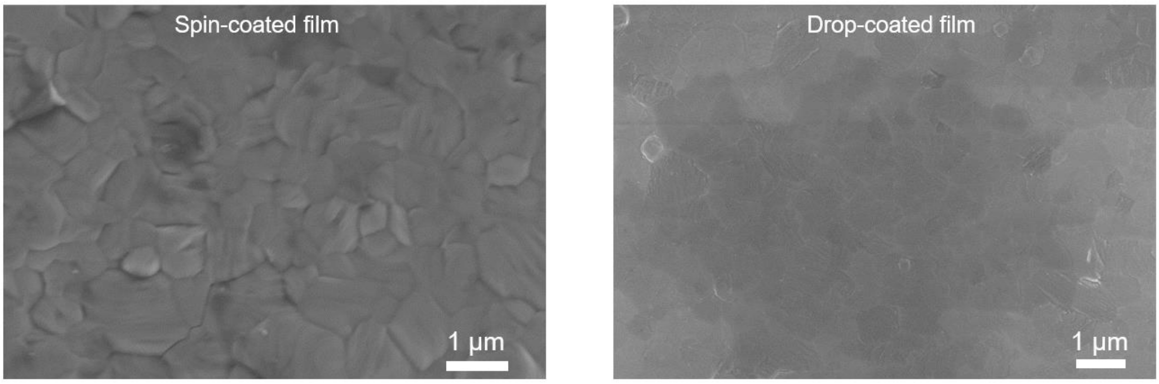

Fig. S3 SEM images for inorganic perovskite films made by spin-coating and drop-coating

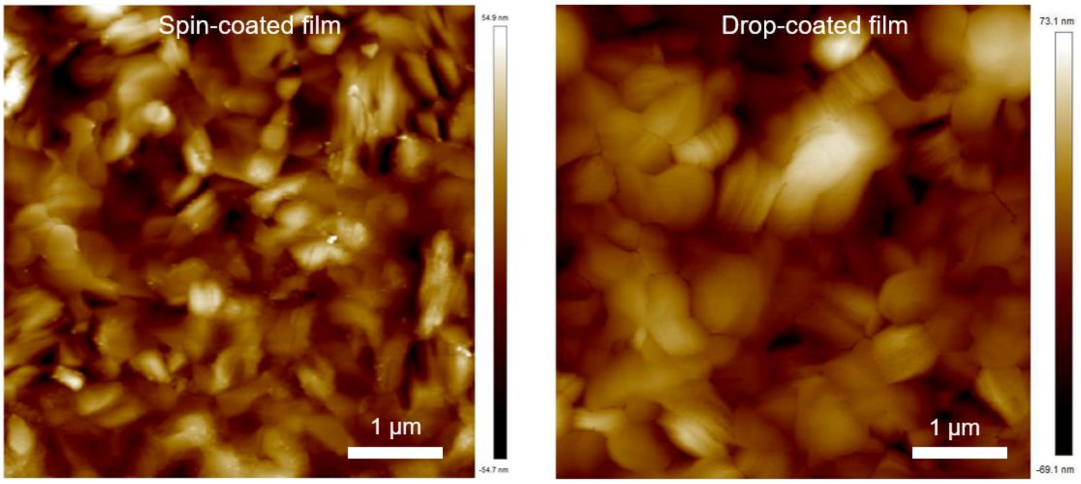

Fig. S4 AFM height images for inorganic perovskite films made by spin-coating and drop-coating

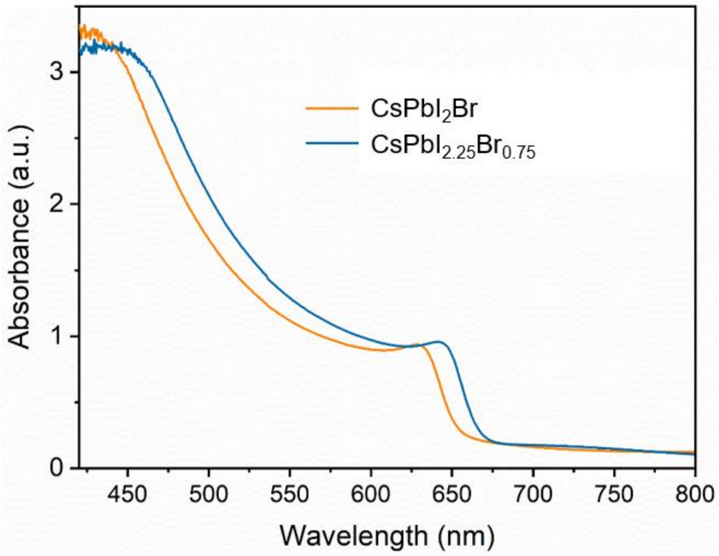

Fig. S5 Absorption spectra for $CsPbI_2Br$ and $CsPbI_{2.25}Br_{0.75}$ films

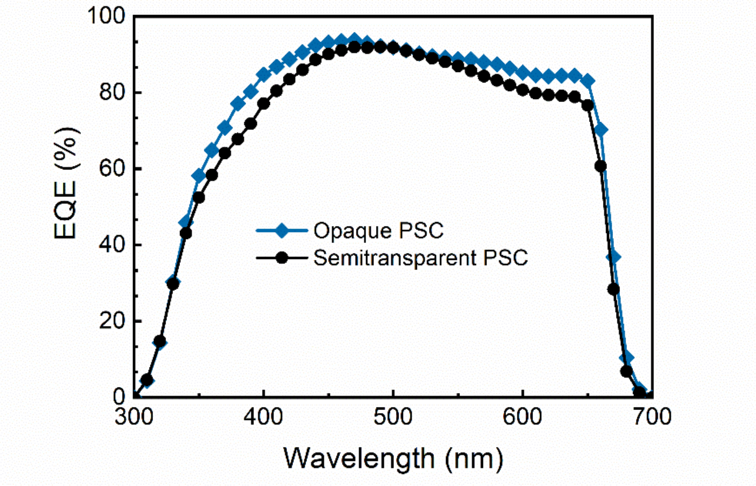

Fig. S6 EQE spectra for the opaque and semi-transparent $CsPbI_{2.25}Br_{0.75}$ solar cells made by drop-coating

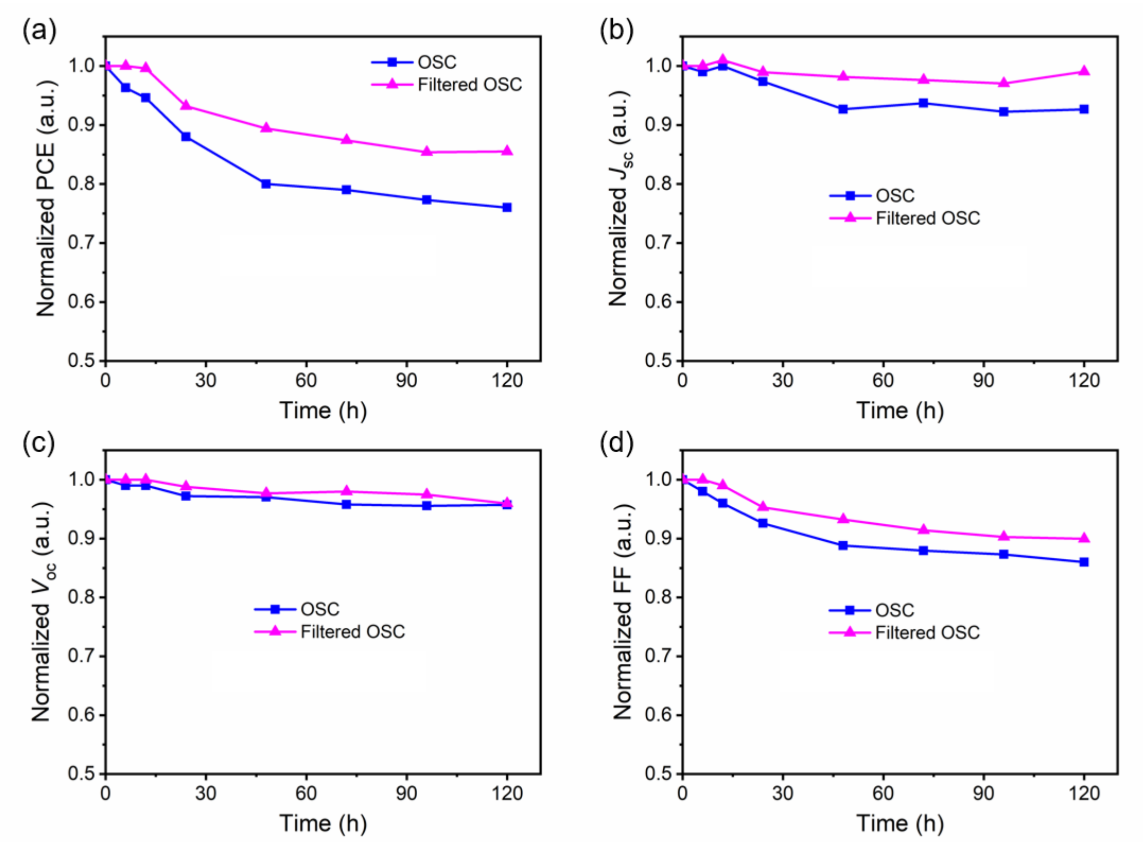

Fig. S7 Performance data for stand-alone OSC and filtered OSC under continuous 1-sun illumination

Table S1 Performance of semi-transparent $CsPbI_2Br$ solar cells made by using $CsPbI_2Br$ with different thicknesses

Thickness [nm]	V_{oc} [V]	J_{sc} [mA cm^{-2}]	FF [%]	PCE [a] [%]
357	1.26	14.22	69.37	12.41 (12.17)
308	1.26	14.08	70.71	12.55 (12.23)
271	1.26	13.90	73.99	12.99 (12.68)
233	1.27	13.43	74.57	12.71 (12.35)

[a]Data in parentheses stand for the average PCEs for 10 cells.

Table S2 Performance of organic solar cells made by using D18-Cl-B:N3:$PC_{61}BM$ film with different thicknesses[a]

Thickness [nm]	V_{oc} [V]	J_{sc} [mA cm^{-2}]	FF [%]	PCE[b] [%]
143	0.84	27.24	77.52	17.65 (17.41)
129	0.84	27.65	77.80	18.00 (17.84)
118	0.84	27.37	78.60	18.17 (17.88)
106	0.84	26.89	78.57	17.82 (17.50)

[a] D18-Cl-B:N3:$PC_{61}BM$ ratio: 1:1.4:0.2 (w/w/w); blend solution: 12.5 mg/mL in CF with 0.5% DPE;
[b] Data in parentheses stand for the average PCEs for 10 cells.

Table S3 Performance of organic solar cells (thickness of D18-Cl-B:N3:$PC_{61}BM$ layer: 129 nm) under light filtered by semi-transparent $CsPbI_2Br$ solar cells with different thickness of the $CsPbI_2Br$ layer

Thickness [nm]	V_{oc} [V]	J_{sc} [mA cm^{-2}]	FF [%]	PCE [a] [%]
357	0.82	12.24	78.97	7.93 (7.68)
308	0.82	12.55	78.47	8.08 (7.95)
271	0.82	12.78	78.37	8.18 (8.05)
233	0.82	12.60	78.03	8.04 (7.85)

[a] Data in parentheses stand for the average PCEs for 10 cells.

Table S4 Performance of filtered organic solar cells made by using D18-Cl-B:N3:$PC_{61}BM$ layer with different thickness. The cells were measured under light filtered by semi-transparent $CsPbI_2Br$ solar cells (thickness of $CsPbI_2Br$ layer: 271 nm).

Thickness [nm]	V_{oc} [V]	J_{sc} [mA cm^{-2}]	FF [%]	PCE[a] [%]
143	0.82	12.60	77.47	8.03 (7.87)
129	0.82	12.78	78.37	8.18 (8.05)
118	0.82	12.66	79.35	8.26 (8.13)
106	0.82	12.32	80.04	8.12 (7.95)

[a] Data in parentheses stand for the average PCEs for 10 cells.
